# Experimental and numerical investigations of microthermal actuator employing the amplification theory for achieving competitive performance

**DOI:** 10.1038/s41598-025-11763-8

**Published:** 2025-07-25

**Authors:** Mohamed Abdelsalam Mansour, Mustafa M. Elsayed, Alaa M. Ali, Abdelrahman Toraya, Noha Gaber

**Affiliations:** 1 Zewail City of Science and Technology, Giza, 12578 Egypt; 2 Université Marie et Louis Pasteur, CNRS, Institut FEMTO-ST (UMR 6174), Besançon, France

**Keywords:** Amplification theory, Micro-fabrication, Hot-cold arms actuator, Micro-actuator, Micromachining, Thermal actuator, Characterization and analytical techniques, Mechanical engineering

## Abstract

The past decade has seen the rapid development of microthermal actuators designs; this is due to their wide usage in various sectors such as biomedical applications and communication. This study presents the experimental assessment, fabrication, and numerical simulation of a novel thermal actuator device applying the amplification theory to achieve competitive overall performance. The device consists of two L-shape lever and half-bridge amplification mechanisms accompanied by microthermal actuators forming all a compliant system. The input displacement is amplified at the output by about 3.55 as a multiplication ratio. Experimental characterization has been performed over a wide voltage range reaching 15 V and achieving 19 μm actuation. Considering how the material’s properties change with temperature and their effect on the simulation results has been proven critical upon comparing experimental with the numerical results. The simulation has shown consistency with experimental results only when employing temperature-dependent models up to a voltage of 12 V achieving 12.9 μm actuation, unlike assuming constant parameters which is widely used in literature that shows noticable deviation throughout characterization range. Additionally, when designing an effective micro electrothermal actuator, there are other parameters that need to be considered besides high output displacement. Therefore, the comparison with other designs has included all the main specifications that are of concern. The performance is discussed based on the main features of any thermal actuator: displacement, temperature, area, and applied voltage, all combined in a performance evaluation index (PEI). The importance of this index is that it evaluates the overall effectivness of a thermal actuator, whereas one can have excellent performance for one of aforementioned features at the expense of the others, which may degrade the total assessment. Our device shows the highest value of this index of 0.0021 μm/mm^2^/K/V at applied voltage of 10 V mapping to the lowest temperature profile at 617.5 K and smallest area among its credible counterparts.

## Introduction

Scaling-down devices have proven to improve many aspects of their performance. For mechanical actuators, it improves not only efficiency and accuracy, but also speed and durability. This can also offer low power consumption with high performance, low cost, design flexibility and easy integration with on-chip electronics^1^. Microthermal actuators have many applications in different sectors such as mechanically tunable photonic crystal lenses^2^, micromirrors^3^, brain implantation^4^, biomolecule manipulation^5^, micropumps, and microtweezers^6^. Additionally, they are extensively used in radio-frequency microelectromechanical systems (RF-MEMS) for the switching process^7^ as data transmission rates in modern communication systems continue to climb into advanced stages, so switching solutions with high speed and demanding performance are currently developing^8^.

The principle of microactuators is to use electrical energy to produce mechanical displacement or force output. There are different types of microactuators: electrostatic, piezoelectric, magnetic, and thermal. Thermal actuators provide maximum force with minimum applied voltage and higher displacement in comparison to the other kinds. Thermal actuators use the mechanisms of expansion and contraction by thermal induction for creating motion. This mechanism is due to the change in the bond lengths between the atoms in a solid material because of the energy increase of the populations of phonons and electrons within the solid. Therefore, injecting or withdrawing heat can be used to create actuation depending on mechanical constraints and elastic deformation. Main mechanisms for heat transmission in thermal actuators are conduction, convection and radiation as a result of ohmic heating or electromagnetic radiation^9^.

Microthermal actuators can be divided into three main types: hot-and-cold arms, chevron, and bimorph. The main difference between them is the mechanism of achieving the actuation. The asymmetric thermal expansion between the hot arm -and- the cold arm -which are the actuator’s components in the first type- is the base of its operation. While the chevron actuator is composed of a bent beam of a single material that expands causing a movement limited to one direction. The bimorph actuator, on the other hand, is based on the structural materials’ different coefficients of thermal expansion^10^. Previous research showed that combining chevron and asymmetric thermal actuators is a promising approach for achieving longer distance with the same voltage compared to using either a chevron only or hot-and-cold arms only. These promising devices are distinguished with a small footprint and low power consumption^11^.

Achieving satisfying displacements with low applied voltages and low power consumption has been extensively studied by many researchers to find the best performance, whether with employing thermal actuators or any other actuation type. One of the approaches used is the amplification theory. The two types of displacement amplifiers that are widely employed are the lever-type and bridge-type^12^. Lai and Zhu^13^ magnified the output displacement of a piezoelectric actuator with a high amplification ratio, where two L-shape lever-type and one bridge-type mechanism are coupled. It was observed that the displacement amplification principle worked perfectly; however, it needed high voltage and a huge footprint. In another study, for the sake of increasing the stroke of the actuator, Pan et al.^14^ developed nonlinear characteristics of the displacement amplifier by altering the length ratio of the compliant section over the rigid segment. To compare between vertical and horizontal motion, Balavalad et al.^15^. investigated the difference by using simulation analysis on U-shape actuator. It was shown that horizontal actuation is larger than the vertical one by one order of magnitude. The actuator simulation was based on Peltier Effect; by applying incommensurate voltages on a loop with two junctions, temperature is produced due to the flowing current causing actuation^16^. These studies indicate that the displacement amplification of thermal actuators is a promising method, and more research is needed to develop more actuators using this approach. However, there is a need to develop micro actuators based on single and cheap materials to make the fabrication process easier and more cost-effective.

In a previous work done by our group, a novel design of a thermal actuator based on double asymmetric two parallel arms was proposed to achieve high stroke actuation according to the amplification theory. The introduced design was studied by numerical simulation using COMSOL Multiphysics^®^ tool. The simulations expected that the displacement can reach 6.01 μm with applied voltage of 7 V. The presented optimized design had many advantages such as giving high amplification with low applied voltage, in addition to being a totally monolithic, joint-less, and compliant system. Moreover, it had a small footprint compared to the electrostatic actuators and the induced temperature did not exceed 563.8 °C^17^.

The aim of this study is to present the fabrication and testing of this previously proposed device and compare its experimental testing with theoretical prediction. In addition, numerical simulations are conducted for more enhancement and optimization of the performance of the device, taking into account realistic physical effects that arise upon conducting the actual operation. Finally, the device is put in comparison with literature in the process of overall performance checking.

## Theory of operation

**Fig. 1:**
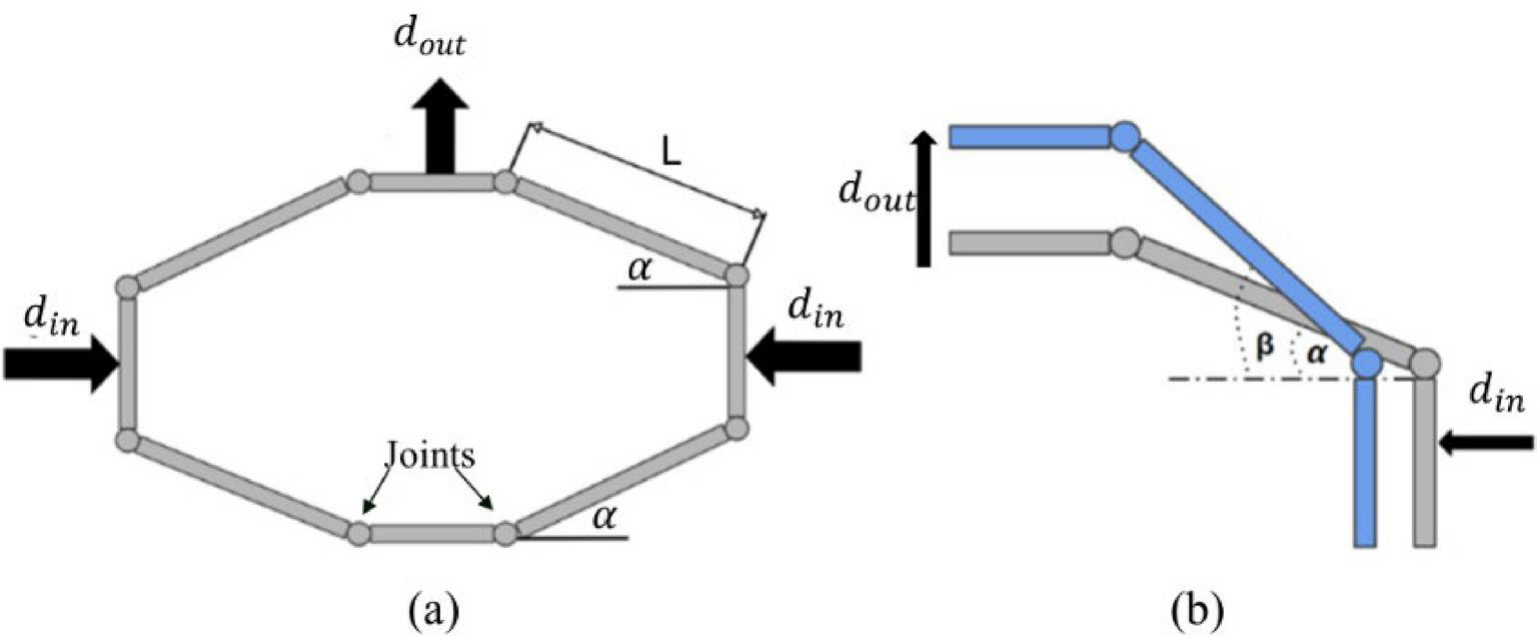
Schematic diagram of the design with rotation joints, (**a**) the whole structure, (**b**) the quarter model. Adapted from^18^.

The term ‘Amplification’ can be used solely when basically referring to increasing the output compared to the input in terms of magnitude. The amplification theory is mostly used in compliant systems with deformable structure forming one piece. The core of the theory is amplifying the input quantity at the output; where this quantity may be force, displacement, or torque. It is applied to systems with limited range of motion. The whole device in our work acts as a half-bridge amplifier, its theory is depicted in Fig. 1 where it shows the input is amplified and translated to the output through the arm $\:L$. According to N. Lobontiu and E. Garcia^18^, based on the symmetry of the architecture shown in Fig. 1(a), the analytical model can be applied on one quarter of the architecture as shown in Fig. 1(b).

The displacement in horizontal and vertical direction can be governed by using the following equations:

1 $$\:L\:cos\alpha\:-{d}_{in}=\:L\:cos\:\beta\:.$$

2 $$\:L\:sin\alpha\:+{d}_{out}=\:L\:sin\:\beta\:.$$

Equation (1) characterizes the horizontal displacement and (2) characterizes the vertical displacement where $\:{d}_{in}$ refers to the input displacement, $\:{d}_{out}$ refers to the output displacement, α refers to the initial angle between the arm and the horizontal line before motion, and $\:\beta\:$ is the angle between the arm and the horizontal line after the motion.

By squaring both (1) and (2) and adding them together to eliminate $\:\beta\:$, the result becomes:

3 $$\:{{d}_{in}}^{2}+2L\:sin\alpha\:\:{d}_{out}-2L\:cos\alpha\:\:{d}_{in}+{{d}_{out}}^{2}=0$$

From (3) the output displacement can be represented by the following equation:

4 $$\:{\:\:\:\:\:\:d}_{out}=\sqrt{{L}^{2}{sin}^{2}\alpha\:+{d}_{in}(2L\:cos\alpha\:-{d}_{in})}-L\:sin\alpha\:.\:\:\:\:\:\:\:\:\:\:\:$$

Therefore, the amplification can be represented as the output displacement divided by the input displacement as shown in (5):

$$\:\:\:\:\:\frac{{d}_{out}}{{d}_{in}}=\frac{\sqrt{{L}^{2}{sin}^{2}\alpha\:+{d}_{in}(2L\:cos\alpha\:-{d}_{in})}-Lsin\alpha\:}{{d}_{in}}.\:\:\:\:\:\:\:\:\:\:\:\:\left(5\right)$$

## Actuator design

The point of interest in our work is integrating L-shape lever mechanism into half-bridge mechanism, which are considered compliant systems that amplify the displacement. Combining L-shape lever-type and half bridge-type mechanisms is a promising technique to amplify output and reduce other problems like unnecessary lateral forces and bending moments^13,19^.

**Fig. 2:**
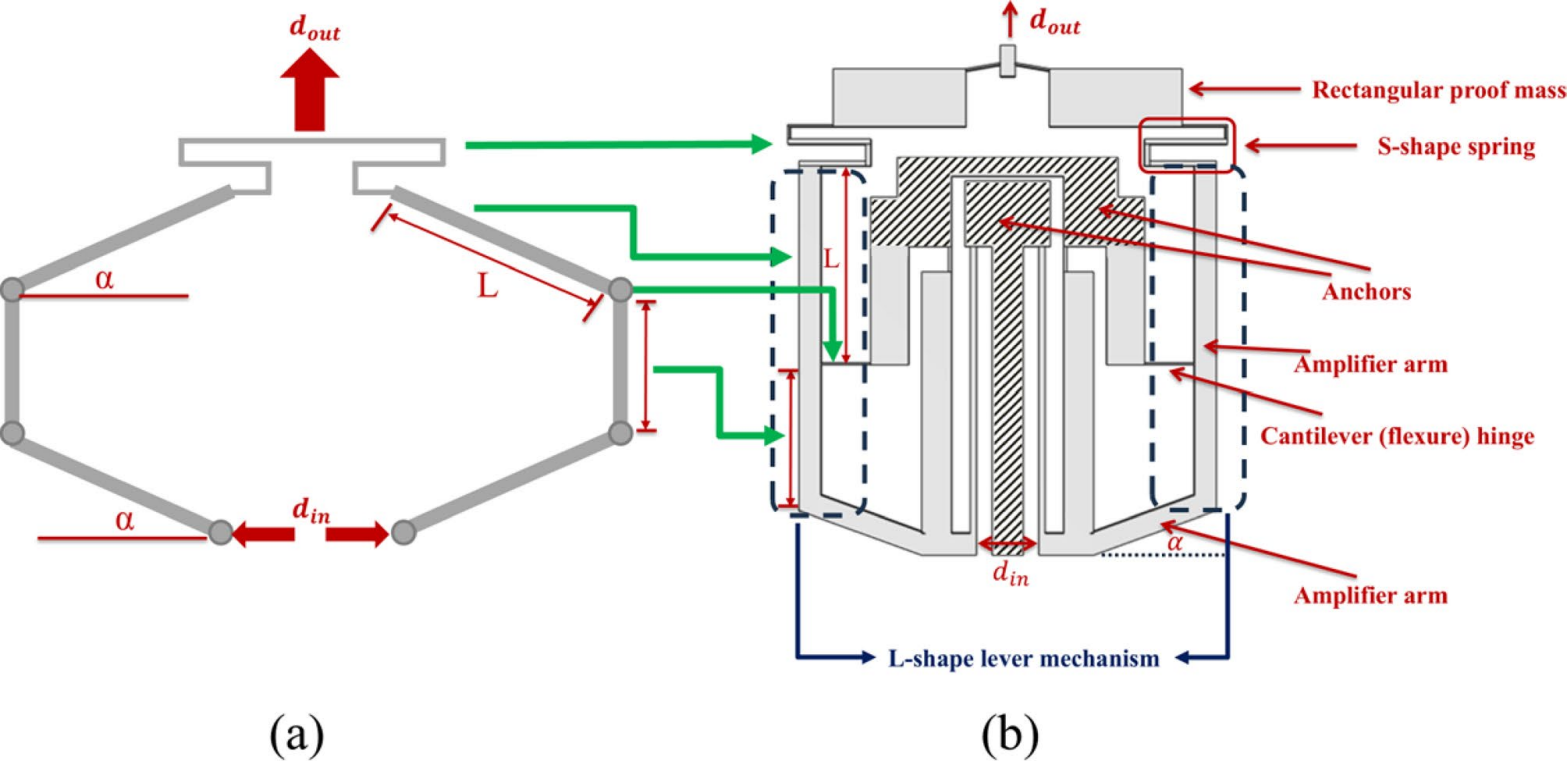
(**a**) A schematic indicating the modifications done on the basic theory depicted in Fig. 1(**a**) to illustrate the modified half bridge mechanism. (**b**) Schematic diagram of the proposed design.

The L-stage amplification mechanism is a compliant lever structure used to convert small input displacements into larger output displacements. It consists of flexure hinges that allow the amplifier arms to bend and connect to the anchors. They act as compliant (flexure) hinges, enabling elastic rotation without mechanical joints. Another part is arm L, where the stage uses one or more connected lever arms and flexure hinges (pivot) to achieve high mechanical gain without requiring additional external components^20^. A modified version of Fig. 1(a) is shown in Fig. 2(a). It helps to understand the transient modifications between the basic theory and our design described in Fig. 2(b). First, we removed the upper joints in Fig. 2(a) to make the system compliant, and added the S-shape spring to couple the displacement. Second, the input displacement is coupled from inside by two hot-cold arms thermal actuators. Two L-shape lever mechanisms which are arranged in mirror symmetrical distribution are used as the stage to amplify the displacement of the thermal actuators and send it to the output. The similarities appear in Fig. 2(b) where the cantilever hinges act as a replacement to the second upper joints to simplify bending and transition of motion. The output displacements of the two L-shape lever mechanisms are then the input to the S-shape spring and proof mass. The main parameters and the integrated lever mechanism of arm $\:L$ are indicated in Fig. 2(b). The dashed blue rectangles show the L-shape lever mechanism while the green arrows show the mapping between different parts. Also, the hatched parts indicate the positions of the fixed anchors. The parameter α affects directional motion and rotational stiffness, while $\:L$ defines mechanical gain. Lever arm $\:L$ transmits displacement from the input side and amplifies it by leveraging the arm length ratio, where flexure hinges provide compliant rotational points allowing the lever to pivot without rigid joints—these are critical to enabling smooth motion and amplification.

**Fig. 3:**
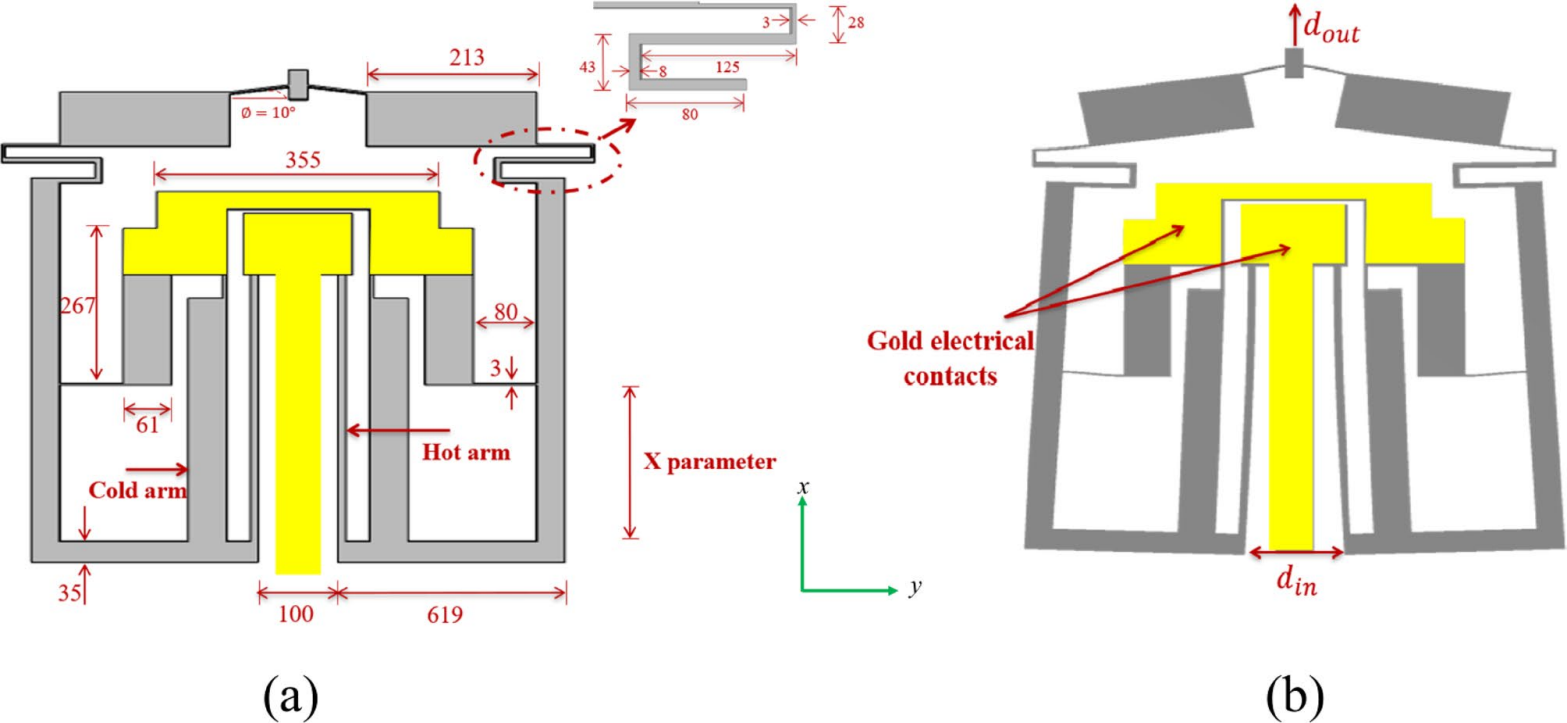
Schematic diagram of the presented asymmetric thermal actuator design. (**a**) The device before applying voltage, indicated its dimensions. (**b**) The device after applying the input voltage on the actuators’ anchors causing hot arms’ expansion to induce the overall stroke.

The device design in this study is shown in Fig. 3(a) where the initial angle α between the arm L and the horizontal line is forced to be zero because it decreases the overall output. The relation between input and output displacement is not straight forward described by Eq. 4 because the device is incorporated with S-shape and proof mass as additional enhancements, which their effect needs to be considered in the equation formulation. Such analysis is not trivial, so we will rely on numerical simulations and experimental investigation instead.

The two actuators have two anchors for both mechanical support and electrical input conduction: a common anchor for the two hot arms, and another one for the cold arms. The cold arm consists of two segments, the main one is a long wide segment of length 420 μm and width 48 μm, while the other one is a short thin flexure segment of length 40 μm and width 5 μm, that connect the main segment to the anchor allowing it to bend easily upon. On the other hand, the hot arm is long and thin with length 460 μm and width 10 μm. Gold was deposited on the anchored parts for better electrical connections. When the input voltage is applied, the hot arm is heated more by ohmic heating effect due to its higher electrical resistance than the cold arm, causing its expansion. Consequently, the actuator rotates giving maximum displacement at the y-axis direction $\:{d}_{in}$. So, to get displacement in the x-axis direction the amplifier arm is connected with cantilever hinges from both sides. The hinge width is very small (3 μm) to give high flexibility during rotation and maximize the displacement in the x-direction $\:{d}_{out}$. Then the folded spring with S-shape acts like a rotating hinge to transfer the displacement from the amplifier’s arms to the proof mass. Finally, the proof mass gives the amplified displacement after maximizing the rotation in the x-direction (Fig. 3(b)). To find the device with the best performance; different designs have been proposed with different X parameter lengths in Fig. 3 (240, 270, 300, 330, and 360 μm). It is worth noting that an adequate analytical model requires complex mathematical methodologies and many considerations to be taken, and still there will be some deviation with the experiment. Pan et al.^14^ discussed the same issue for a basic compliant system. He took some of these factors’ effect into consideration and made a comparison between simulation results, nonlinear analytical model, and experiment, which still gives some error due to other reasons he suggested. Instead of a complex analytical modeling that may not be very accurate, we will rely on numerical simulations to optimize the X parameter length.

## Methodology

**Fig. 4:**
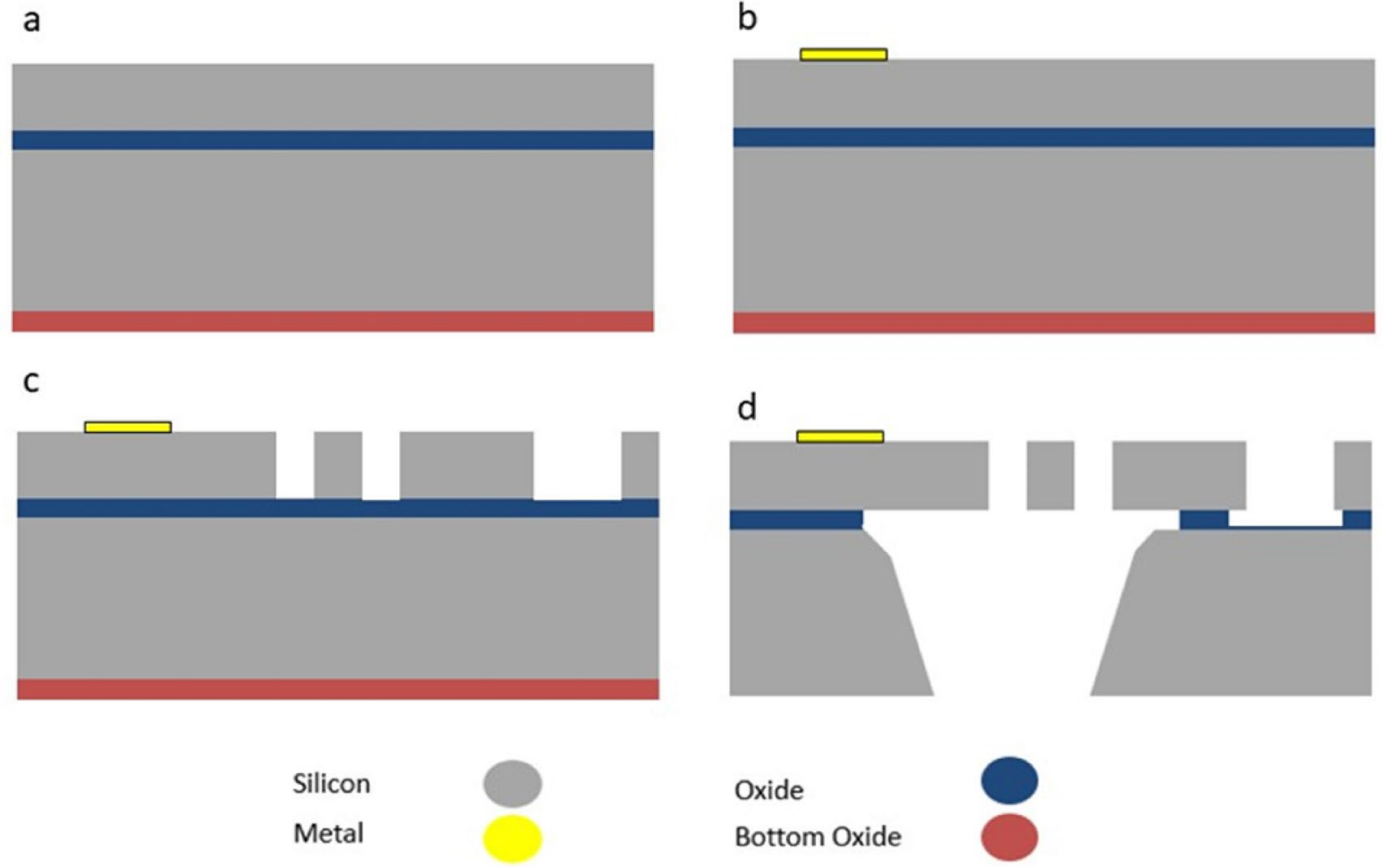
Illustration of the main fabrication steps of the thermal actuator using SOIMUMPS. (**a**) The starting SOI wafer with bottom oxide layer. (**b**) The deposition of pad metal. (**c**) Patterning the structure layer. (**d**) Releasing the structure using back etching. Adapted from^21^.

The device has been manufactured by the standard SOIMUMPs process as part of the MUMPs^®^ service. Masks layouts design for the intended device has been drawn using CleWin layout editor software. Then, the design was sent to MEMSCAP^®^ company for its realization. Figure 4 shows the main fabrication steps of the device. Briefly, the process consists of 4-masks for patterning and etching. Silicon on insulator (SOI) wafer with a 25 μm silicon thickness (active layer), 2 μm oxide thickness, and 400 μm substrate thickness was used for the fabrication process. There is a Bottom Side Oxide layer initially exists on the starting substrates to protect the non-patterned areas of the substrate from etching. The first mask was used to conduct a liftoff process to pattern a metal stack of a 20 nm layer of chrome and a 500 nm layer of gold to act as the conductive terminals of the device. Using the second mask, the upper silicon layer is patterned by lithography to give it the shape of the device, which was then etched using deep reactive ion etching. The following step is the back etching for the bottom silicon till reaching the bottom oxide layer. Then, the Buried Oxide layer in the back etched regions was removed using a wet etching process to release the structure. More details about design rules and consideration can be found in SOIMUMPs handbook^21^. The structure incorporated some holes to allow the liquid etchant to reach the oxide layer for an efficient removal of the buried oxide layer in the areas to be suspended. After receiving the dies from the manufacturer, the suspended structures were released in our facility using a 40% (V/V) HF solution for 1.5–2 min. Then, the dies were rinsed with deionized water, followed by isopropyl alcohol to avoid the stiction of the suspended structure with the substrate.

The thermal actuator has been experimentally tested using PLV50 Probe Station manufactured by CascadeMicrotech^®^. Two probes have been contacted on the two anchors. The input voltage has been applied on one of the probes, while grounding the other one. The applied voltage ranged between 5 and 15 V with a step of 1 V. Using a CCD camera attached to a viewing microscope, snapshots for the actuator have been taken before and after applying each voltage. The displacement corresponding to each applied voltage has been measured from the snapshotted images using the image processing open-source software ImageJ, the values have been taken with respect to a fixed point in the snapshots.

Simulations have been conducted on COMSOL Multiphysics^®^ simulation tool by finite element analysis. Three physics modules have been coupled to integrate the working conditions of the device. Solid mechanics module has been used to model mechanical deformation and define anchors as fixed constraints. Electrostatic-current physics module has been used to pass electric current inside the actuator’s arms by defining one anchor as input applied voltage and the other as the ground. This current induces heat to flow within the actuator, whose effect is simulated by the role of heat transfer in solids module, which is responsible of modeling the thermal expansion and heat transfer in the active layer. The heat transfer coefficients have been set to be 3,200 W/m^2^. K for both gold and silicon; this value has been deduced from the experimental results reflecting the influence of environmental conditions on the actuator. The applied voltage has been swept to configure the displacements obtained and temperature profiles. A figure of merit (FOM) has been defined as the ratio between the output displacement and the device area. To find the device with the best performance, a sweep has been performed on the X parameter in Fig. 3 from 240 μm to 360 μm with step of 30 μm. At each point, the displacement has been extracted and FOM has been calculated.

## Results and discussion

**Fig. 5:**
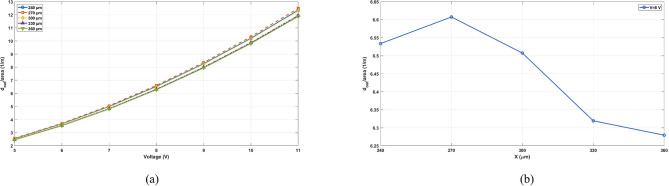
FOM evaluation: (**a**) Comparison of the values of the FOM ($\:{d}_{out}$/area) versus the applied voltage for different values of X parameter, (**b**) FOM plotted as a function of X under a constant voltage value.

**Fig. 6:**
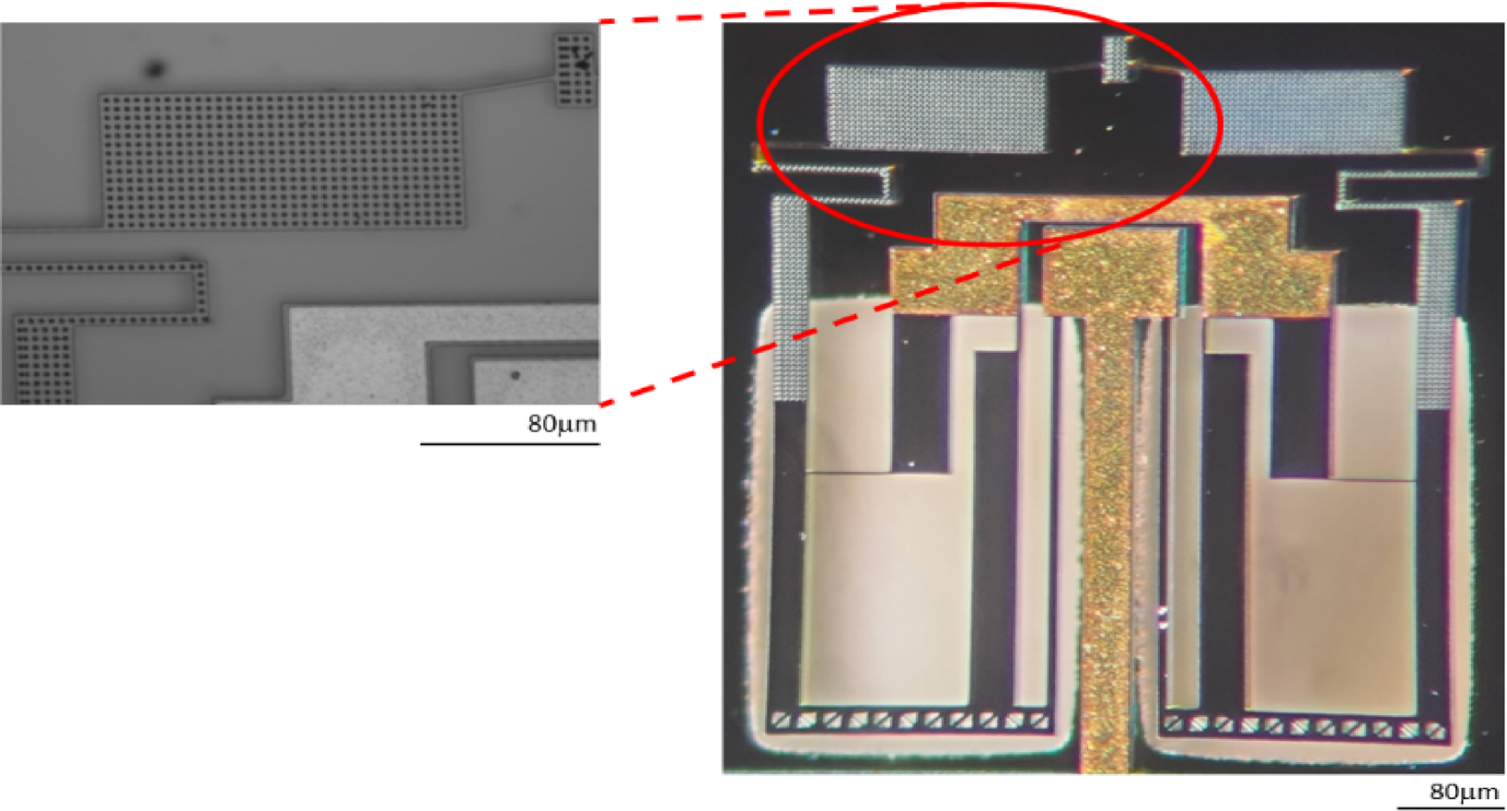
Microscopic image of the fabricated device with X parameter equal 270 μm. Inset: Zoomed-in view showing the holes incorporated for achieving a thorough release by wet etching.

In the process of compromising the area with the actuation displacement using the numerical simulation based on the aforementioned FOM, the study has included a sweep on X parameter to find the best performance. It showed that the maximum displacement increases with increasing X, but the footprint also increases with X, which is undesirable for microsystems. The FOM (that is defined as $\:{d}_{out}$/area) has been calculated for different values of X as presented in Fig. 5. It has been used primarily for intra-study comparisons, that is to compare the same device but with different parameter values. Since the voltage range and temperature profile are almost the same for all devices, it is desirable to identify the best value that maps to highest amplification with minimum area. As noticed from Fig. 5(a), the highest curve corresponds to the value X = 270 μm that renders the device’s footprint to be 706 × 818 µm^2^. Figure 5(b) illustrates the relationship between FOM and X under constant voltage. The device at X = 270 μm is shown in Fig. 6 after its fabrication. It shows the two thermal actuators connected to the two levers. These two levers are connected to two S-shapes for delivering the output amplified displacement to the tip. The golden areas in the figure show the gold electrical connections. The inset shows the holes that were incorporated to permit the wet etchant to reach the oxide layer for releasing the structure. Moreover, experimental tests have been conducted on this device as it has the best FOM.

**Fig. 7:**
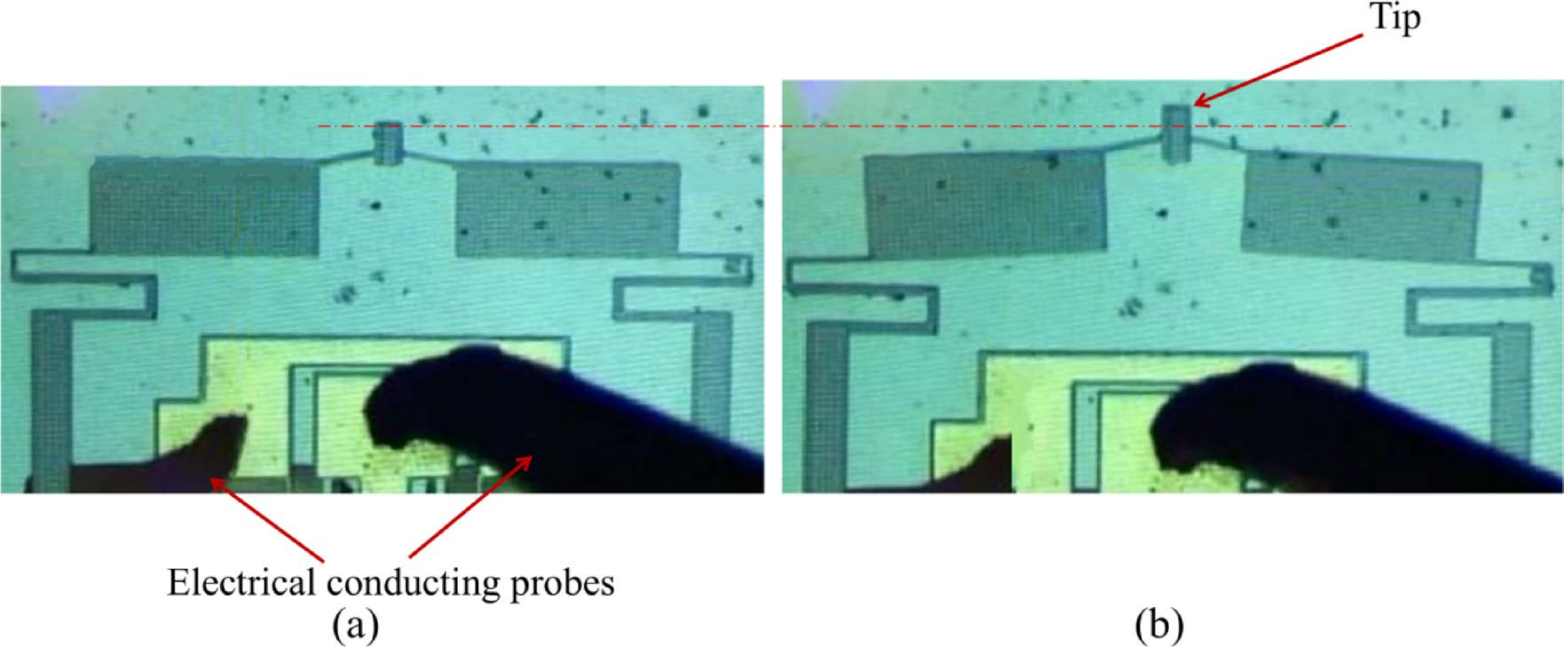
Microscopic image of the device: (**a**) at stationary condition, (**b**) after applying the input voltage showing the actuation happens. The red dashed line is a reference bar to illustrate the displacement of the tip.

Figure 7 shows a working device before and after actuation (a and b respectively) under the microscope. It shows the deformation of S-shapes delivering the amplified displacement to the tip upon actuation. Actuation is achieved by applying voltage on the anchors (which are also the electrical electrodes) using the probe station, causing the hot arms start to heat up and expand more than the cold arms leading to lateral displacement. This displacement transfers to the hinges to give vertical displacement that reaches the S-shapes, which act as a rotating hinge. The hinge deforms and transfers the amplified output displacement from the two sides to the tip. The amplification ratio has been numerically calculated by dividing the output vertical displacement by the input lateral displacement from one sided hot-and-cold arms across the operational range, which resulted in an averaged value of 3.55 with a +/- deviation of + 0.0216/−0.0181, which are the deviation from the average for the lowest and highest values.

**Fig. 8:**
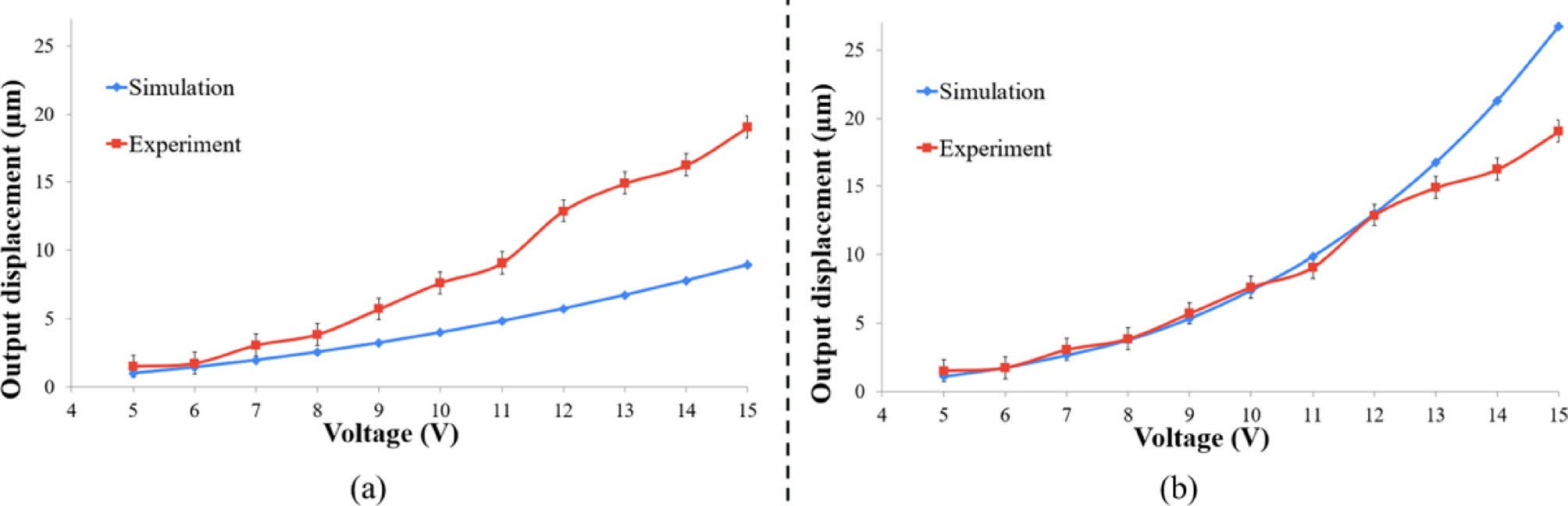
Simulation and experimental results of the maximum output displacement versus the applied voltage: (**a**) when assuming the materials characteristics are constant, (**b**) after taking the materials characteristics temperature dependence into consideration.

The experimental results of the device under test show the corresponding $\:{d}_{out}$ achieved at each applied voltage. The measurements are allocated within error of + 0.823 μm and − 0.771 μm from the depicted values. By taking the mean of multiple measurements of displacement, the error values are the deviation of the highest and lowest displacement values from the mean. This margin is indicated by the error bars in Fig. 8. It has been then compared to those obtained from numerical simulations. When the values of all materials’ characteristics were put as constants in the COMSOL simulation -as done in several literature- there has been an incredible deviation between the extracted and simulation values that can be seen in Fig. 8(a). This can be attributed to the high temperature effect on intrinsic properties of silicon. In fact, this issue has been widely emphasized in literature due to its critical impact on modeling accuracy. It stems from the temperature-dependent behavior of material properties and their effect on the discrepancy between numerical and experimental results. Additionally, assigning appropriate heat transfer parameters remains also a nontrivial challenge in many designs^22–24^.

The basic contributor to the temperature behavior has been the heat transfer coefficient. It is well-known that this coefficient depends on many complicated conditions such as the thermal properties of the ambient medium, the hydrodynamic characteristics of its flow, the thermal boundary conditions, and the geometry and surface roughness of the device surface. Even in literature, its value is given as a range, not as a model or certain values. Thereby, one cannot predict an exact value for it, except through experimental results. So for our work, it has been chosen based on trial and error by comparing the experimental results to the simulated ones until reaching the value that leads to similarity between the two behaviors. However, heat transfer was reported extensively before that -at the microscale- it is predominantly dominated by conduction. The role of convection is relatively small but still important to consider^25–27^.

For the different Silicon properties, we had to find models or data as a function of temperature to investigate if their change with temperature is significant. By inserting their temperature variation in the simulator and comparing the numerical results to the experimental ones, some of them have been found necessary and others have been neglected. Taking the thermal expansion coefficient (TEC) as a start, its values have been taken from experimental results presented before in literature^28,29^. Since the device has been operated at a wide range of temperatures, which corresponds to the applied voltages range, the contribution of TEC has been found necessary to be taken into consideration during the numerical study.

The second factor that has been investigated is the Young’s modulus. Its effect has been studied by inserting its values corresponding to different temperatures into the simulator. Such values have been obtained from a graph introduced by Z. Liu et al.^30^. The simulation results showed that Young’s modulus does not affect the output displacement significantly (it only changes it by 0.03%) so we proceeded with the simulations by considering its value as constant in all cases. Similarly was the analysis of density effect; it has been found that it changes by maximum 0.8% with temperature and has no effect on simulation results. So it was put as a constant, and the modelling was according to^31^. Heat capacity has been considered also based on data in^32^. It also doesn’t affect the simulation outputs in the least. On the other hand, the main influence comes from the thermal conductivity ($\:K$) that has been modeled in the simulation as a function in temperature based on (6)^33^,

6 $$\:K={K}_{0}[1-B{\left(T-{T}_{0}/T\right)}^{A}]$$

where $\:{K}_{0}$ is the thermal conductivity at room temperature, $\:T$ is the actuator temperature, $\:{T}_{0}$ is the room temperature, $\:A$ and $\:B$ are constants and by curve fittings their values were chosen to be 0.8705 and 1.093 respectively^33^. As shown in Fig. 8(b), when implementing (6) in the simulator a great agreement between the experimental and numerical results has been achieved at a wide range of voltages till 12 V.

Electrical conductivity also has a huge role as the electrical resistivity has an exponential dependence on temperature, as shown by (7)^34^,

7 $$\:\rho\:=A{e}^{{E}_{G}/2kT}$$

where $\:A$ is a constant, $\:k$ is Boltzmann’s constant, $\:T$ is the temperature in degrees Kelvin, and$\:{E}_{G}$ is the band gap. The values of $\:A$ and $\:{E}_{G}$ are obtained from a fitting of the experimental data according to the doping concentration^34^. Therefore, this experimental model depends greatly on the doping concentration. In our case, the doping concentration is not uniform. According to the manufacturing process: “The top surface of the Silicon layer is doped by depositing a phosphosilicate glass (PSG) layer and annealing at 1,050°C for 1 hour in Argon”^21^. Unfortunately, a model for electrical conductivity at such gradient doping conditions couldn’t be found in literature to our best knowledge. Hence, as an approximation, its value was put as a constant of a high value (80 kS/m) near the surface until the diffusion depth of 0.8 μm, where the doping concentration is high; and a low value of the bulk silicon (100 S/m) at the remaining of the structure, where the doping concentration is much lower. These values were deduced based on SOIMUMPS given sheet resistance and diffusion conditions that enabled us to estimate approximate values for the diffusion depth and the conductivity. However, putting the electrical conductivity as constant is believed to be one of the main reasons for values deviation at voltages higher than 12 V (Fig. 8(b)). On the other hand, fabrication tolerances that affect both the dimensions and material properties of the device also contribute to this discrepancy.

**Fig. 9:**
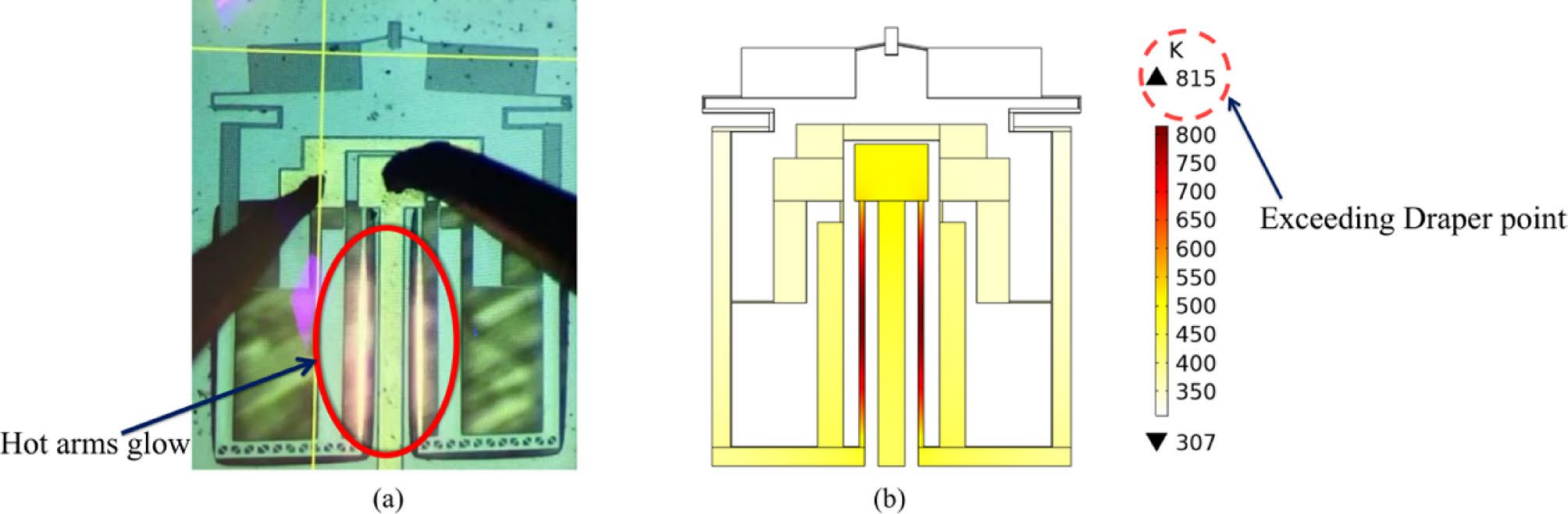
The glow behavior in the hot arms as a response of high voltage (12 V): (**a**) the real device under the microscope, (**b**) the numerical simulation of the temperature profile at input voltage of 12 V showing the spot of maximum temperature and its value.

The maximum applied voltage has been limited to 12 V because the experimental performance of the device at higher voltage does not agree with the expected one from the simulation, as discussed before. Moreover, the device starts to glow at 12 V as shown in Fig. 9(a), especially at the hot arms where the highest temperature occurs. That glow is believed to be due to the electromagnetic radiation emitted covering the visible range. To investigate this behavior, the device has been simulated at the same voltage. The temperature profile at 12 V clarifies the areas of maximum temperature profile reaching 815 K, and hence maximum thermal resistance Fig. 9(b), which agrees with the Draper point principle that states that almost all solid materials visibly glow as a result of blackbody radiation at 798 K^35^. So, in some scenarios it may be convenient to limit the maximum applied voltage to 11 V at which the temperature is only 707 K.

**Fig. 10:**
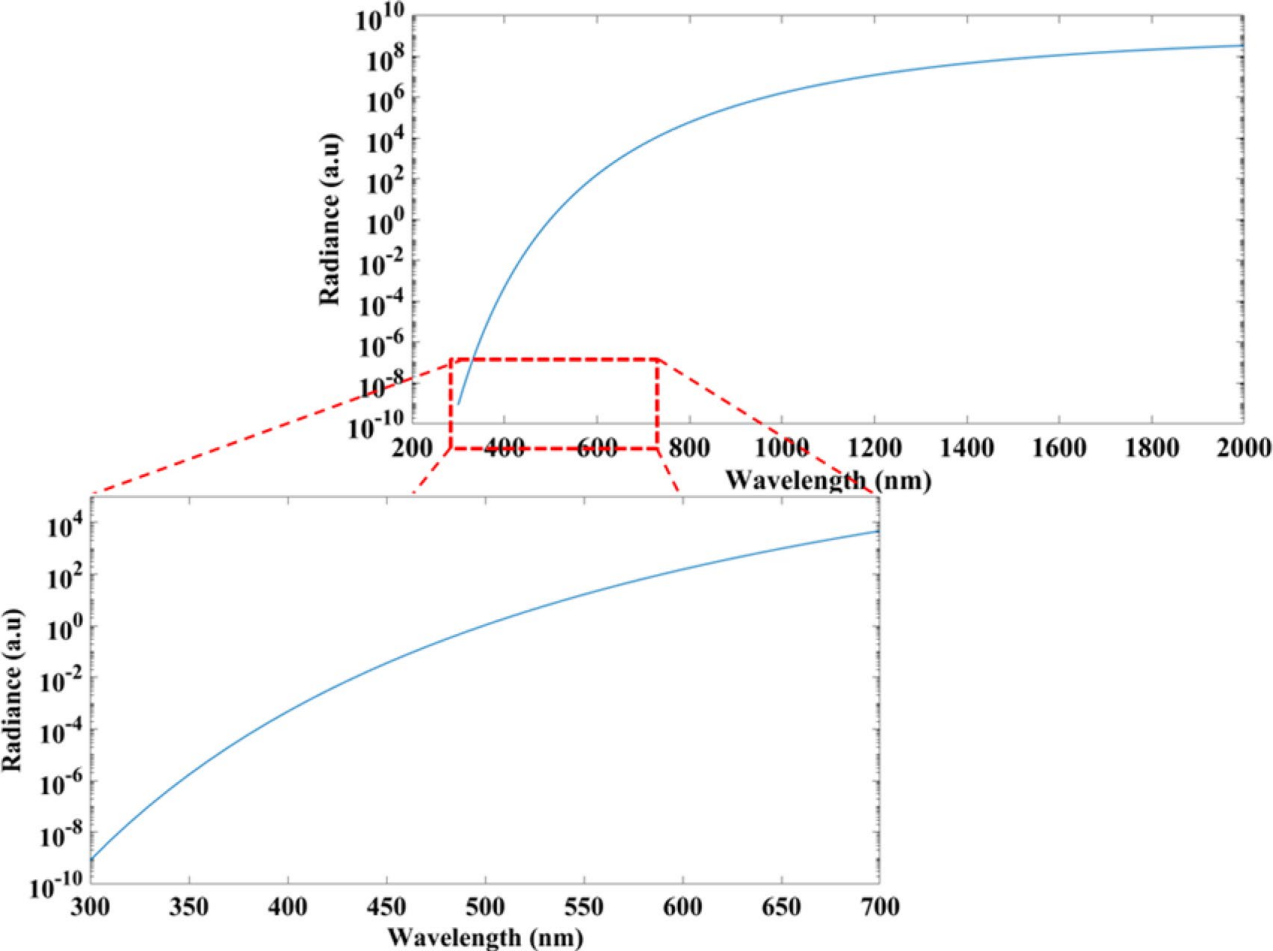
Silicon radiation at temperature 815 K versus the light wavelength.

To estimate the emitted spectrum from the hot parts of our device, Planck’s law for blackbody radiation has been enhanced by multiplying the energy $\:{L}_{BB}$ radiated per unit volume by silicon emissivity $\:{\epsilon}_{Si}$ which is about 0.6 in the visible range. The effect of doping and device thickness is negligible at high temperatures^36^. By applying Planck’s law for silicon as following, we roughly can estimate its spectrum,

8 $$\:{L}_{Si}={\epsilon}_{Si}\:{L}_{BB}={\epsilon}_{Si}\frac{2h{c}^{2}}{{\lambda\:}^{5}({e}^{\frac{hc}{\lambda\:kT}}-1)}$$

where $\:{L}_{Si}$ and $\:{L}_{BB}$ are the spectral radiance of silicon and blackbody respectively, $\:{\epsilon}_{Si}$ is silicon emissivity, is the emitted wavelength, $\:h$ is the Planck constant, $\:c\:$is the speed of light in vacuum, $\:k$ is the Boltzmann constant, and $\:T$ is the absolute temperature of the body. The log scale of the spectrum is plotted in Fig. 10. When applying 12 volts, the temperature resulted from our COMSOL simulation is 815 K. By inserting this value in the above equation, the spectrum of silicon shows a substantial radiation at the visible range, maximized at 700 nm (visible red), as depicted in the inset of Fig. 10, which appears as a glow during the experiment.

To evaluate the overall performance of our device, we have benchmarked the performance of our microthermal actuator against counterparts documented in the literature, considering key performance metrics such as displacement (d), operating temperature (T), applied voltage (V), and device area (A). The favor for actuator devices is achieving large displacements with minimal footprint, while operating at lower voltages and temperatures. To encapsulate these parameters, a performance evaluation index (PEI) is employed, defined as d/A/T/V. We have extracted the relevant voltage range reported in each referenced work, along with the corresponding displacement and temperature values. We then compute the PEI across this range and report the maximum PEI value observed, as it reflects the peak performance potential of the device under its tested conditions. Table 1 represents the comparison between the maximum attainable value of this index for different designs in ascending order multiplied by 1,000 to simplify the comparison between the values. There is no direct trend of PEI change with voltage, the general case is that the behavior of a microthermal actuator is not linear. It is worth noting that the maximum value of PEI (mentioned in the table) is not necessarily obtained at the maximum displacement; since that at high voltages the temperature does not increase linearly, so it could show a rapid incline trend which decreases the index value.

**Table 1:** Comparison of the performance of our device with other microthermal actuators found in literature.

		Ref.	Material	Displacement d (µm)	Temperature T (K)	Area A (mm x mm)	Applied Voltage V (V)	Performance Evaluation Index (PEI) d/A/T/V (µm/mm^2^/K/V)$\:\times\:$1000
Simulation only		^37^	Silicon	22	317	9.9$\:\times\:$9.03	2	0.39
		^38^	Silicon	1.13	462	1.3$\:\times\:$0.316	10	0.6
		^11^	Silicon	4.8	650	0.4467$\:\times\:$0.653	6	4.22
		^39^	Silicon	22.55	404	1.92$\:\times\:$2.01	3	4.82
		^40^	Silicon	8.12	1,400	0.31$\:\times\:$0.134	8	17.45
			Silicon	4.85	780	0.34$\:\times\:$0.134	6	22.75
			Silicon	4.82	600	0.41$\:\times\:$0.134	6	24.37
			Silicon	5.06	670	0.38$\:\times\:$0.134	6	24.72
		^41^	Silicon	2.22	335.53	0.664$\:\times\:$0.3	1	33.21
		^42^	Aluminum	1.1	303	0.37$\:\times\:$0.83	0.02	591.07
		^43^	Aluminum	10.94	721.15	0.66$\:\times\:$0.102	0.2	1,126.72
		^44^	Aluminum	17	473.8	0.895$\:\times\:$0.32	0.09	1.393
Experimentally validated	Simulation parameters not sufficiently temperature dependent	^45^	Silicon	2.7	660	0.4$\:\times\:$0.4	7	3.65
		^46^	Silicon	1.11	608.1	0.21$\:\times\:$0.273	6	5.31
		^47^	Nickel	30	1,114.5	1.4$\:\times\:$0.5	1	38.45
		^48^	Silicon	5.3	1,020	0.0415$\:\times\:$0.267	7	66.99
		^49^	Nickel	173.46	470	2.413$\:\times\:$1.314	1	116.4
	Simulation parameters varying with temperature	^50^	Silicon	12.041	768	1.65$\:\times\:$0.508	10	1.87
		^51^	Silicon	128	635	5$\:\times\:$5	9	0.9
		^52^	Silicon	24.7	634	4.5$\:\times\:$0.85	8	1.27
		Our design	Silicon	7.616	**617.54**	**0.818** $\:\times\:$ **0.706**	10	**2.14**

The comparison reveals that the different designs exhibit a wide range of PEI values, based on whether the achieved displacement comes on the expense of large area, large voltage, high temperature, or all. Designs in ‘Simulation only’ section relied solely on simulations without adequately accounting for how temperature variations impact the devices’ key parameters. It was common to see most material properties assumed as constant in these computational analysis, a simplification that often misses the nuances of real-world behavior. For instance, reference^11^ adopted only a basic first-order approach to model the temperature dependence of resistivity. Furthermore^37,41^ have used a second-order approximation of resistivity with temperature, however this is still not enough, especially when there is no experimental result to compare with. This oversight can lead to unexpected and unrealistic high index values, as in the rest of the section, and significant discrepancies between simulated and experimental results, such as depicted in Fig. 7(a).

In the ‘Experimentally validated’ section there are two subsections: The first one, ‘Simulation parameters not sufficiently temperature dependent’, is about designs with experimentally measured displacement, but simulated temperatures that are not modeled with the materials’ properties sufficiently led to deviation of simulation from testing results. The plotting of experimental and simulation results in^45^ shows the same trend of Fig. 7(a), this is because it used resistivity only as a first-order approximation dependance on temperature, and the rest of material properties were put as constants. B. Barazani, S. Warnat, and T. Hubbard^48^ went a step further; they modeled thermal and electrical conductivity across different temperatures, bringing their experimental and simulated results much closer together. However, some gaps still remained, due to not modeling all the relevant properties. The thermal actuators in^47,49^ show the same deviation problem because of inaccurate modelling with temperature variations. They are also fabricated out of nickel. Despite its functionality, it is inherently more vulnerable to corrosion and mechanical degradation over time than silicon-based alternatives, shows high temperature profile at low voltages, and in some cases may not be compatible with silicon industry. V. Ionescu^48^ took a different track, attempting to bridge the gap between experimental and simulated results of another study by tweaking material property values. This strategy, while well- intentioned, didn’t fully address the issue as it still relied on constant values throughout the simulation, but used different values from those proposed in the original paper. The original study based its values on specific parameters from a particular fabrication program. In contrast, Ionescu’s values were either derived from an alternative source with different fabrication protocols and conditions, or from another simulation that also relied on static parameter values. This deviation raised questions about the consistency and applicability of his simulation results compared to the original experimental data.

Moving to the second subsection, ‘Simulation parameters varying with temperature’, the results in^50^ have a very small deviation between experiment and simulation compared to the previous sections in the table. Even though its modelling of material’s properties was more accurate than the previous designs in the table and supported by measuring temperature experimentally, the authors were aware that this small deviation is due to other factors such as fabrication tolerance, and not modelling all the temperature-sensitive properties. Then there is^51^, which stands out for its comprehensive approach. The team not only measured temperature and displacement experimentally but also ran simulations considering how temperature change affects material properties like thermal conductivity and expansion coefficient, the values were close especially in the displacement curve. The values of these parameters were in good agreement with their correspondence in our paper. This holistic approach not only aligns well with our study but also confirms the importance of considering temperature effects in microthermal actuator designs. H. Zhu et al.^52^ has also taken care of this point drastically leading to a very good matching between experimental and theoretical assessment. Their research has been careful to model each parameter adequately. Thermal parameters and electrical parameters were addressed separately indicating the importance of each one, their comparison with other literature was clearly mentioning this point that whether the compared reference used temperature-dependent thermal/electrical parameters or not. Their research aligns with our discussion about considering the effect of temperature on the unavoidable critical change in the parameters of materials. This helps in keeping the results consistent, and in supporting logical explanation of the behavior of devices. Although their research shows careful analysis and consistency in results, the PEI index of our design is better, as indicated in the table. In comparison to the other devices in this subsection, our device achieves the best PEI, the lowest temperature, and the smallest area.

## Conclusion

In this study, the experimental testing of a novel design for an optimized microthermal actuator employing the amplification theory has been introduced, along with its numerical simulations. Simulations done employing constant parameters such as TEC and thermal conductivity showed deviation from experimental results. So, considering the dependence of these parameters with temperature is found to be important to accurately characterize the device. Employing such temperature dependence has then led to a very good agreement across a wide range of applied voltages. The maximum actuation reached -that is in consistence with simulation- has been 12.9 μm upon applying input voltage of 12 V with a maximum produced temperature of 815 K. However, since this temperature is higher than the Draper point -at which the glowing occurs- it may be preferable to limit the working range till 11 V at which the actuation is 9 μm and maximum temperature is 707 K only. The overall performance is discussed while taking into consideration the main characteristics of thermal actuators, which are the output displacement, temperature, footprint, and voltage applied. A performance assessment has been evaluated in this context by calculating the PEI values which have a maximum value at 10 V for our design. Conclusively, the device is put in comparison with various designs in literature commenting on the credibility of our analysis and its outstanding performance with its peers.

## Data Availability

All data generated or analysed during this study are included in this published article.
